# Exploring barriers to adherence to COVID-19 infection control measures and advice among immigrants in Norway: A qualitative study

**DOI:** 10.1016/j.jmh.2024.100292

**Published:** 2024-12-25

**Authors:** Prabhjot Kour, Gunnar Saebo, Kristin Buvik, Janne Scheffels, Øystein Vedaa, Thor Indseth

**Affiliations:** a Pandemic Centre, Department of Global Public Health and Primary Care, University of Bergen, Årstadveien 17, 5009 Bergen, Norway; b Department of Alcohol, Tobacco and Drugs, Norwegian Institute of Public Health, Myrens verksted 2, 0473 Oslo, Norway; c Fafo Institute for Labour and Social Research, Postbox 2947 Tøyen, 0608 Oslo, Norway; d Department of Health Promotion, Norwegian Institute of Public health, Zander Kaaesgate 7, 5015 Bergen, Norway; e Department of Psychosocial Science, University of Bergen, Christies gate 12, 5015 Bergen, Norway; f Department of Health Service Research, Norwegian Institute of Public Health, Sandakerveien 24C, 0473 Oslo, Norway

**Keywords:** COVID-19, Infection control measures and advice, Adherence, Immigrants, Norway

## Abstract

**Background:**

Since the beginning of the COVID-19 pandemic, various infection control measures and advice have been issued by different authorities across the world to prevent the spread of the infection and associated mortality. However, the support for and ability to follow recommendations varies across countries and populations. In Norway, studies have shown that immigrants bear a higher burden of COVID-19 infections, hospitalizations, and associated mortality compared with the general population. This warrants investigation into how people from different backgrounds understand and act in response to COVID-19 measures and advice.

**Aim:**

This qualitative study explored the experiences and perceptions of immigrants in respect of attitudes and barriers to adherence to COVID-19 measures and advice.

**Methods:**

In-depth interviews were conducted with immigrants from nine different country backgrounds. Data was analyzed using thematic analysis.

**Results:**

The analyses reflected barriers at three different levels: individual, cultural, and systemic. At the individual level, language, information barriers and material barriers formed sub-themes. At the cultural level, expectations relating to collectivistic cultural practice emerged. At the systemic level, poor translation and late dissemination, and overload and frequent modification of information were evident.

**Conclusion:**

This study provides enhanced understanding of how and what immigrants perceive as barriers to adherence to COVID-19 measures and advice. Understanding immigrants’ perspectives is crucial to tailoring policies and improving information dissemination among different groups in the future.

## Highlights

• Individual, cultural, and systemic barriers to adherence to COVID-19 measures and advice were identified.
• Cultural expectations and practices significantly impacted adherence to COVID-19 measures and advice.
• Issues with poor translation, delayed dissemination, and frequent changes in guidelines were noted.
• Understanding immigrants' perspectives is key for tailoring policies and enhancing adherence in diverse immigrant groups.

## 1 Introduction

After being declared as pandemic by the World Health Organization on March 11th, 2020, the COVID-19 disease has become one of the most important public health crises in modern history (WHO 2020). Since then, countries around the globe have employed containment strategies to rein in the pandemic by means of infection control measures and advice. These strategies are non-pharmacological interventions that include behavioral changes in social and individual routines, namely hand washing, use of face mask, hygiene practices, avoiding physical agglomeration and keeping social distancing, and implementing quarantine or isolation on suspicion or confirmation of COVID-19 (Ferguson et al., 2020, Villela, 2020). In Norway, during the pandemic, the infection control measures and advice that apply have varied, depending on local or national infection rates.

Greater adherence to the infection control measures and advice has been shown to reduce mortality. An increase in mortality of 81.3% was found in countries with low adherence rates, that is, the United States, Sweden, Poland and Russia, in contrast with 8.4% in countries with high adherence rates, that is, Germany, France, Spain, and the United Kingdom (Margraf et al., 2021). Several factors, depending on the context, may influence the population's adherence to infection control measures and advice, including perception of government response efforts, general health information source, social media source, knowledge of COVID-19 (Al-Hasan et al., 2020), self-perceived risk, higher perceived benefits, desire to protect others (Yang et al., 2020, Mehanna et al., 2021), seriousness of COVID-19 in terms of high morbidity and mortality, negligence, unemployment, livelihoods (Abeya et al., 2021) and trust in authorities (Madar et al., 2021).

Further, socioeconomic status and health inequalities strongly predict the morbidity and mortality of the COVID-19 disease (Cockerham, 2021). This pandemic has put a great toll not only on health and healthcare systems but it has also exacerbated socioeconomic differences in health (Devakumar et al., 2020, Devakumar et al., 2020). Several countries have reported barriers to adherence and a higher burden of COVID-19 infection and hospitalization rates among immigrants as compared to the general population (Bhala et al., 2020, Rostila et al., 2021, Niedzwiedz et al., 2020, Yancy, 2020). This tendency also appears to be stronger in high-income countries (Hayward et al., 2021). A study conducted in the UK reported that Black and south Asians (especially from Pakistani ethnicity) were more likely to test positive with COVID-19 compared to white British. Moreover, those who were socioeconomically deprived and had no academic qualifications were hard hit, which could be explained by area-based deprivation, overcrowded accommodation, high-risk occupations, and pre-existing chronic health conditions such as asthma, diabetes, hypertension, in addition to barriers to access the healthcare (Niedzwiedz et al., 2020, Hayward et al., 2021); and the authors assume that these factors may be the cause of insufficient adherence to infection control measures and advice (Niedzwiedz et al., 2020). Similar findings of health and socioeconomic inequalities have also been reported in comparison studies between immigrants and the general population conducted in the US (Yancy, 2020), Sweden (Rostila et al., 2021), and Denmark (SSI 2020).

Inequalities have also been reported in Norway among immigrants from certain countries in terms of overrepresentation, hospitalization, and associated mortality from COVID-19 (Indseth et al., 2021, Indseth, 2021). Results from the Norwegian County Public Health Survey of COVID-19 suggest that immigrants have higher self-reported adherence to infection control advice on hygiene than the general population (Nilsen et al., 2021). Yet, the response rate among immigrant groups in that survey was low. In addition, closer examination of the individual behaviors showed that those with an immigrant background (i.e., from low-income countries) had lower adherence to refraining from shaking hands and avoiding public transport (Aarø et al., 2021). Furthermore, a Norwegian register study highlighted that the differences in socioeconomic and previous health conditions has limited explanatory value for explaining the differences between immigrant groups (Labberton et al., 2022). In light of these opposing findings from quantitative studies, as well as the fact that, for various reasons, it is difficult to reach the immigrant population with such population-based surveys, there is a need to study how people in Norway with immigrant background perceive and have experienced COVID-19 infection control measures and advice using a qualitative approach, to gain a deeper knowledge and understanding of this group's situation. This is especially important considering Norway is a multicultural society of significant ethnic diversity (Oppedal et al., 2005) and that 18.5% of the Norwegian population are immigrants (migrated themselves and Norwegian-born to immigrant parents) (SSB 2021). Further, there is growing literature about the impact of COVID-19 communication and its effectiveness for different immigrant groups (Maldonado et al., 2020, Koval et al., 2021). A scoping review identified various approaches used in highly affected countries to ensure adherence to infection control measures. These included social media engagement, science-based policy communication, strategic narrative control, nonverbal communication, ideologically influenced messaging, and the use of metaphors and storytelling (Mohamed Nour and Kisa, 2024). In countries such as France, Germany, Italy, and Portugal, government communication emphasized self-protective behaviors, transparency, positive messaging, and citizen engagement, avoiding fear-based tactics (Jacob et al., 2023). Additionally, effective strategies involved two-way communication and tailoring messages to specific demographics (Jacob et al., 2023). European Union institutions also played a significant role, using platforms like Twitter to share clear and consistent information during the vaccination campaign, counter misinformation, and engage with the public (Jiménez-Alcarria and Tuñón-Navarro, 2023). These examples highlight the critical role of transparent, engaging, and tailored communication, and we argue that Norwegian findings should form a part of this global picture.

In this study, we explore how immigrant groups from nine different country backgrounds in Norway have experienced and perceived barriers to adherence to COVID-19 infection control measures and advice.

## 2 Methods

A qualitative approach was implemented to gain knowledge about the attitudes and experiences of immigrants in respect of the barriers they face when adhering to infection control measures and advice. An exploratory qualitative design was used to obtain a comprehensive description of the factors acting as barriers to adherence.

### 2.1 Recruitment

The Norwegian Institute of Public Health (NIPH) hired a research consultancy firm, Opinion, to recruit the participants and to perform interviews. The participants were selected using a purposive sampling strategy, informed by the concept of information power as outlined by Malterud (Malterud et al., 2016). This approach ensured the recruitment of individuals who could provide relevant data based on their direct experiences with COVID-19, and infection control measures. The country-backgrounds represented in the sample were chosen due to their significance to the research objectives, as these immigrant groups were among the largest in Norway and disproportionately affected by COVID-19 in terms of infection rates and hospitalizations (T. Indseth et al., 2021, Indseth, 2021).

Opinion recruited 55 participants through a combination of established dialogue and network groups and advertising on Facebook and other social media platforms. More than half of the sample, 28 participants, were recruited by snowballing via networks. Five participants were recruited via contact persons in various organizations and three were recruited by the moderator randomly stopping people on the streets of Oslo. Men and women over the age of 18 years from different country backgrounds, either born outside of Norway or with at least one parent born outside of Norway, were included in the study. Snowball recruitment, in particular, proved effective in reaching individuals who might otherwise be difficult to reach, offering a diverse and meaningful sample for the study.

### 2.2 Participants

A total of 55 participants from nine different country backgrounds were included (Appendix 1, Table 1). We deliberately tried to include participants from the country backgrounds with the largest infection and hospital infection rate in Norway (Indseth et al., 2021). Seven were from Afghanistan, and six each from Bosnia/Serbia, Iraq, Pakistan, Poland, Somalia, Sri Lanka, and Turkey, 31 were women and 24 were men. There was a large age range among the participants: from 21 to 77 years old. The length of residence in Norway varied between 1 to 48 years, and 7 participants were born in Norway. Some of the participants were working in health services, kindergartens, transport, restaurants, IT, education, or their own businesses, others were laid off from their jobs due to the pandemic and a few were students or pensioners. Although language proficiency in Norwegian also varied among participants, most interviews were conducted in Norwegian implying good understanding and knowledge of Norwegian language. Further, the living situations of the participants varied considerably from small apartments to large houses.

### 2.3 Data collection

The data was collected through 54 in-depth interviews between March and April 2021. Fifty-three were individual in-depth interviews (Kvale and Brinkmann, 2009). One dyadic interview (Morgan et al., 2013) was conducted with two participants. Twenty-six interviews were conducted by phone, 27 were conducted digitally via Teams and the dyadic interview was conducted physically. Two moderators from Opinion conducted the interviews, which were designed to take 60 min. In practice, they lasted between 30 and 60 min. Five interviews were conducted in the mother tongue of the participants using a professional interpreter, while 2 of 54 interviews were conducted in English and the remainder in Norwegian. The interview guide consisted of open-ended questions, focusing on how the COVID-19 pandemic had affected their everyday life, and their knowledge and awareness of the infection control measures and advice, including quarantine and isolation advice. The interview guide was created by researchers working in the field of migration and health at NIPH and Opinion. All the interviews were audio recorded and transcribed, 52 into Norwegian and 2 into English, by a team from Opinion. Authors KB, GS and JS participated as observers in about 1/3 of the interviews. They communicated with the interviewers from Opinion on chat-messages during the course of the interviews and were thereby able to probe and add supplementary questions.

### 2.4 Data analysis

The interview transcripts were read thoroughly by the authors, and data was analyzed using thematic analysis as outlined by Braun and Clarke (Braun and Clarke, 2006). The analysis was conducted inductively, meaning themes emerged directly from the data without the use of predefined frameworks, allowing for a nuanced understanding of barriers to adherence to infection control measures. The process began with familiarization, where the authors (PK, KB, JS, and GS) repeatedly read the transcripts to gain a deep understanding of the data and identify potential patterns. After this, PK conducted a systematic line-by-line reading of the transcripts, generating initial codes that captured key elements related to participants' experiences with COVID-19 infection control measures and advice. These codes were generated inductively, ensuring that they directly reflected the content of the data.

Once the initial codes were identified, all the authors engaged in collaborative discussions to refine and agree on the coding process. This allowed for consensus-building and ensured that the data interpretation was consistent across the research team. The codes were then grouped into broader categories, leading to the creation of potential themes and sub-themes. This phase involved careful consideration of Braun and Clarke's criteria of internal homogeneity (ensuring coherence within themes) and external heterogeneity (ensuring distinctiveness between themes). As themes began to take shape, the team revisited the data multiple times to ensure that the themes accurately reflected the full scope of the data and that no important codes had been missed during the initial coding process.

As the analysis progressed, we noticed recurring themes with no new patterns emerging, leading us to conclude that thematic saturation had been reached. To ensure the themes were comprehensive and accurately reflected the participants' experiences, we re-read the entire data set and validated the identified themes and sub-themes against the raw data. Finally, the themes and sub-themes were organized into a coherent framework, accompanied by illustrative quotations from participants to support the findings. These final themes are presented in the results section, providing a detailed and transparent account of the barriers to adherence to infection control measures and advice during the COVID-19 pandemic.

### 2.5 Ethical aspects

The study was conducted by Opinion, a research consultancy firm hired by NIPH. The research procedure was designed and followed in accordance with the Norwegian Data Protection Authority. The Regional Ethics Committee for Medical and Health Research in Norway has assessed that the topics investigated in this research project fall outside the Health Research Act. The research project therefore does not have an ethical approval from the Regional Ethics Committee in Norway. However, the ethical aspects of the current research project have been assessed by NIPH and found to be acceptable. A data processing agreement between NIPH and Opinion which regulates the data collection and processing of personal data was signed by respective privacy representative and security managers at both NIPH and Opinion. The Data Protection Officer (DPO) at Opinion reviewed and approved the data collection in line with current privacy legislation. The DPO was not in any way involved with the approved research.

Consent from the participants was given orally in the introductory part of the conversation, to ensure that the participant received all the necessary information despite language barriers, an interpreter was used in the interviews that were not conducted in Norwegian. The conversations were recorded by audio and / or videotape, which was stored by Opinion. The interviews were then transcribed and anonymized by Opinion, and NIPH received only anonymized information for processing. The recordings were deleted as soon as the interviews were transcribed and delivered. Only Opinion processed personal information in this project, on behalf of NIPH.

## 3 Results

**Fig. 1:**
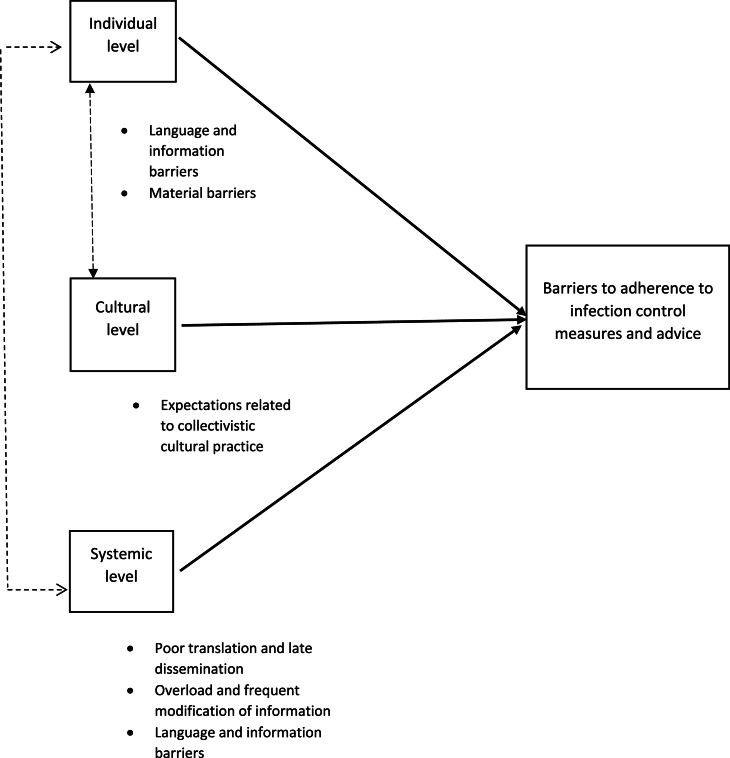
Thematic network of barriers to adherence to measures and advice.

Three main themes were identified while analyzing the data, and each theme had various sub-themes. These themes represented what could be seen as barriers to adherence to COVID-19 infection control measures and advice at three different levels related to the participants’ belief and value system at each level, namely (1) individual, (2) cultural and (3) systemic. A thematic network (Fig. 1) of themes and sub-themes was created to illustrate these barriers. The figure also shows that some themes and sub-themes are intersecting at different levels and this intersection has been discussed in the discussion part below.

### 3.1 Barriers at individual level

Barriers at individual level to the adherence to infection control measures and advice were due to the participants’ individual belief and value systems. The sub-themes that emerged under this level were (a) language and information barriers and (b) material barriers:

***(a) Language and information barriers***

Limited skills in the Norwegian language acted as a barrier to adherence to measures and advice among some participants. Poor local language skills hindered their ability to use the available information about the current measures and advice associated with the COVID-19 pandemic. Those who had a shorter stay of residence in Norway were more likely to have poor language skills, limited social networks and less integration into Norwegian society. One newly arrived participant stated that he had stopped following the Norwegian news on the pandemic and on the current measures and advice because of his inability to understand the language.

*Here in Norway, I lost my connections with the world, in the sense that I don't follow any news, because I don't understand the language. It's hard for me to learn the language without going to school. I came here, and went to school, and the fee was so high that I could not afford it. So, I tried learning by my own, but still, it's a hard language.* (30, M)

Language barriers were also more prevalent among elderly participants, who either needed help from their children to understand the measures and advice, or they often remained unaware of them.

*We planned for one of my children to come with one of the grandchildren to visit, but then I received a phone call, "No, the rules have changed now, so we can't come". But I am not aware of the rules having changed. In itself, it is difficult to keep up with everything and I depend on someone to do it, in a way, to let me understand it.* (64, F)

Some of the participants had some language skills, but still described difficulties in understanding medical/academic terms like infection control:

*"Infection control" [smittevern], I do not understand. Because it's a double word, right? I do know what "infection" means… The infection that is happening now.*

*"Infection control", have you heard that word? (Moderator asks) No.* (26, F)

In addition to poor language skills, limited social networks and less integration within mainstream society among some participants prohibited their access to and understanding of the information about measures and advice and hence acted as a barrier to adherence.

Furthermore, a few of the participants showed a lack of understanding of the seriousness of the COVID-19 pandemic, seemingly dismissing available information, or not interpreting it properly. A few participants mentioned that the disease is associated with mild coughing and that they have not encountered anyone with serious symptoms, and hence it is not perceived as serious enough to keep them home or to get tested or to access information on the measures and advice.

*Not afraid. We and other friends are not afraid of this situation. It´s life.* (60, M)

However, most participants expressed their frustrations over other persons’ (mostly young and elderly) dismissal of the danger of COVID-19 or their non-adherent behavior. And by “other persons”, they meant both people they knew and did not know. As one participant said:

*"Corona is worse than flu," I said, "No, flu is worse." "Corona does not exist, it is a virus just like the flu," he said* (67, F)

*He said to me that "corona is just nonsense and I do not understand why the authorities should intervene so hard." And it frustrated me a little.* (29, M)

***(b) Material barriers***

Material barriers were associated with the living conditions of the participants. These in turn often correlated with the length of residence in Norway, the longer the residence, the better the participants’ living conditions. Several participants reported living in small apartments or houses. Some lived in large families, with different generations in the same house in close proximity. A few participants stated having a single toilet for the large families they live with and hence had less ability to maintain social distancing.

*But it is very difficult to avoid infection if you live in the same small apartment, you touch the same things, you use the same things as the others, so it is difficult. I think that if I had been infected, I would have infected my roommate regardless of whether I had slept in a separate room or not*. (45, F)

Some also mentioned types of housing, where living in apartment buildings in densely inhabited areas implied closer contact with people than in areas where neighbors were more distant:

*It's a bit weird, because this area has been the epicenter in Oslo and the thing is that we live in blocks of flats and more densely. And if one of my nieces and nephews had gotten it* (COVID-19), *then I would have definitely gotten it, too.* (29, M)

In addition to living conditions, several of the participants mentioned being in work situations that require physical attendance and interaction with colleagues and customers. Some were working in frontline jobs where there was continued exposure to and close encounters with others. This included persons working in healthcare facilities, and taxi and bus drivers. Some also mentioned mandatory use of public transport due to lack of affordability of personal vehicles. Hence, they cannot adhere to the measure and advice of social distancing.

*There are many who work as bus drivers or cleaners, and then you are forced to move around* (29, F)

Some also talked about fear of losing work due to economic burden and insecure work contracts. A few participants mentioned the work pressure from employers, whereby the employer did not prioritize infection control in the workplace and instead encouraged them to disregard the measures and advice. One participant who had a part-time job stated that she had been asked to go to work even though she had symptoms that could be compatible with COVID-19, and she had to use public transport to reach the site. Another participant mentioned using the subway to travel to the test center with his sister, as this was the only option they had to travel there.

*Often, if I was sick and called, they [the employers] said no, you must go to work because you work almost alone in the evening, and there would be no one in the office. But they do not understand my health, they just understand that I must go because they have no stand-ins.* (37, M)

### 3.2 Barriers at cultural level

Barriers at cultural level were associated with the participants’ cultural and familial belief and value system that hampered adherence to the measures and advice. The following sub-theme was identified under this main theme, (a) expectations related to collectivistic cultural practice:

***(a) Expectations related to collectivistic cultural practice***

Several participants described family cultures where they spent a lot of time with family and family friends and taking part in these social meetings was a clear expectation. These cultural practices mainly related to participants from non-European backgrounds. They described themselves as family oriented, with large extended families they were close to, and saw regularly, and with whom they consequently practiced less social distancing during the pandemic. They called these social networks their “inner circle”, which included parents, in-laws, siblings, cousins, uncles, aunts, and grandparents.

*Large network, that's how it is… My blood family, it's just an aunt and uncle, but we know quite a few people in the community. There are a lot of people here, so I have aunts and uncles around, female, and male cousins, but also family members of my age* (21, F)

*Family is quite crucial to us; it is something we need to have around us. Parents, siblings, female cousins, male cousins, uncles, aunts, grandparents* (23, M)

In addition to large extended families, some participants described it as typical of their culture that people support and help each other in everyday life and are often mutually dependent on each other's households, including taking care of children and grandchildren. Further, the elderly often sleeps over with children other than those they usually live with, and thus a change in household was a part of their regular lives. For some, social interactions among the immediate families, such as hugging each other, continued as usual during the pandemic. One participant described that her niece visited her several times a week and that they performed many activities together, like eating and playing. Nevertheless, most of the participants asserted that they only met with their closest families:

*There will be no other strangers, it's just our children*. (65, F)

However, several participants observed how this could still be challenging, with large families and a situation where everyone was used to spending time together with the whole family:

*It's difficult. Because if my mom invites my brother, then we must invite another brother and my sister. My sister has 5 children. My brother has 3, another one has 4, plus his wife and… right?* (47, F)

Additionally, a few participants talked about expectations of visiting family gatherings and fear of conflicts within the families. One participant stated that it was difficult to say no to an invitation, so as not to disappoint the family members, and thus it was not easy to point out activities that were contrary to the measures and advice. Another participant described a hierarchical culture where the older generation determines the premises to a greater extent than the younger ones, including for social interactions within the families. One participant described her mother as a person who did not take recommendations seriously and therefore invited too many people home. The participant also mentioned that there was greater awareness of measures and advice among younger people in her community than among the older generation.

*But my parents would not accept me not visiting them at all, even if there is pandemic. It's that reproach: “Even though it's a pandemic, I'm your mom, I'm your dad,” this belief within our families is difficult to overcome and dominates to some extent. They feel sorry if it takes too long [between visits]. Three to four weeks is fine, but if months pass. They've been blaming me: “We haven't seen your girls in months now” and then I say, “Yes, but it's a pandemic”. “So what? We are family, we have to see each other." Even though I try, I have parents who want to see my daughters and meet them, so it's not that easy.* (36, F)

### 3.3 Barriers at systemic level

Barriers at systemic level were related to the participants’ experiences with the health authorities. These experiences at systemic level were divided into two sub-themes, (a) Poor translation and late dissemination of information and (b) Overload and frequent modification of information:

***(a) Poor translation and late dissemination***

Unclear translations and their interpretations into their respective languages were considered a barrier to adherence to measures and advice by several participants. They mentioned that the SMSs sent by the municipality were difficult to understand and perceived the translation as untrustworthy since it was poorly written and contained grammatical, spelling, and semantic errors.

*Yes, we received an SMS, first in Norwegian and then in our mother tongue, that they might have translated on Google translate, it was so poorly written… you can understand it, but there were grammatical errors, punctuation, spelling mistakes, everything…. the municipality does not have a clue about the minority population, not as much as they should have, I think* (24, M)

One participant also added that there is low reach of information in immigrant-specific areas and that the dissemination of information in these areas is poor:

*The authorities were too late to inform, in other languages and be more proactive. Because I said to all my friends to begin with, "OK, now the virus is on the west side of town* [the affluent]*"* (29, M)

***(b) Overload and frequent modification of information***

Some participants stated that advice changed frequently, and the information was too comprehensive and detailed, which made it difficult to understand and adhere to. They perceived the information involving frequent changes to measures and advice as being too vague, entailing ambiguities in understanding the terminology and concepts such as cohort, number of people allowed to visit, what applies to social and private events and seating arrangements.

*My friend says that 200 people are allowed if there are fixed seats and then there was someone else who said "No, that's not allowed, they mean cinemas and cafes and theater", something along those lines, they were not particularly clear about what they meant by that. But also, like, 10 close contacts and so on. I do not think they were necessarily unclear, but sometimes there was so much information out there that it just became too much*. (29, M)

Some participants talked about the lack of instructions in simple Norwegian language. In their opinion, the language of those messages was “too academic” for them to understand. One participant stated that there was complete chaos in understanding the messages, as they were unclear and created doubts. He gave as an example:

*Five people visiting minus those who live in the house, and people in the same kindergarten are counted as the same. No one understood it, so I thought «just stay at home, do not invite anyone”* (21, M)

One participant said the briefing of the press conferences, which she considered as an important source, was difficult to understand as they used academic Norwegian language:

*Like these press conferences, it is a very academic language, there is a lot that we do not understand, because there are many sub-points and unclear* [difficult] *language* (29, F)

Further, some participants mentioned that it was challenging to understand who were considered as close contacts. One participant stated that he hardly meets other people but when he meets other people they tend to belong to different households.

*There are a couple of my partner's friends, and one regular best friend and little brother, two cousins and a good friend that we do not meet so often, but who live close to where I live.* (47, F)

Hence, participants described their confusions in understanding different measures and advice that were frequently changing, and difficulty in taking in all the comprehensive information.

## 4 Discussion

The purpose of this study was to explore the experiences and perceptions of immigrants to Norway from nine different country backgrounds in respect of barriers to adherence to COVID-19 infection control measures and advice. We found that the immigrants seemed to face barriers at three different levels. First, language and information, and material barriers at individual level. Second, having expectations related to collectivist culture stood out as barriers at a cultural level. Finally, participants also reported barriers at the systemic level, which included poor translation and late dissemination of information, in addition to overload and frequent modification of the information on infection control measures and advice. Furthermore, we argue that these three levels intersect to some degree, such that cultural factors influence individual behavior and vice versa. Also, language and information barriers largely intersect at both individual and systemic level. We would also like to point out that while most interviews were conducted in Norwegian and the majority of participants were able to communicate during the interviews, language barriers still emerged as a significant theme, particularly for certain participants. These barriers were not necessarily due to a complete lack of proficiency in Norwegian but rather varying levels of fluency and comfort in using the language, which impacted more complex or nuanced communication.

Previous research has shown that lack of local language skills can act as a major barrier in effective health communication to ethnic minority groups. In addition, with most messages and communication being in the local language, minority groups may feel hindered due to language difficulties (Scheppers et al., 2006). A recent systematic review on COVID-19 among immigrants stated that these groups face challenges in accessing adequate information due to language barriers in high income countries, because of limited language skills and limited availability of translation services (Hayward et al., 2021). Also, limited local language skills were associated with less COVID-19 testing among immigrants (Kim et al., 2020). Similar language difficulties have also been documented among immigrants in Denmark, leading to challenges in understanding the COVID-19 measures and advice and constituting a barrier to adherence, according to one study (Brønholt et al., 2021). These previous findings are in line with those of the present study. Further, it has been documented that those immigrants who arrived in the country most recently and had limited language skills had to face more difficulties in accessing COVID-19 information on infection control measures and advice, which correlates with the experiences of some of our study participants (Hayward et al., 2021). In addition to those with shorter residence in Norway, many elderly participants in our study reported having limited language skills and were either dependent on their children for receiving the information or did not receive it at all. This posed further difficulty for them, as they could not seek digital healthcare when there was no other available alternative (Brurberg and Himmels, 2021). Furthermore, low awareness of the danger of the COVID-19 pandemic has been reported among immigrants in Italy (Ceccarelli et al., 2021), which is similar to our finding in which some participants mentioned that they did not perceive COVID-19 as a serious disease and hence did not adhere to the measures and advice.

A systematic review of what facilitates and what hinders adherence to infection control measures and advice among immigrants indicated that there are several factors that may contribute to a higher risk of COVID-19 and lower adherence (Regmi and Lwin, 2021). These are: living in an inter-generational overcrowded household, a socioeconomic situation where stopping work is impossible due to fear of losing one's job, and an inability to follow the recommended measures and advice (Niedzwiedz et al., 2020, Yancy, 2020). They are also often employed in frontline jobs and were more likely to work outside their homes during the lockdown period, with resulting low adherence and increased risks of catching COVID-19, which may then spread to family members in a crowded household (Rostila et al., 2021, SSI 2020, Regmi and Lwin, 2021). Furthermore, Devakumar and colleagues state that 44% of the medical workforce in the UK consists of Black, Asian and minority groups, who are treating COVID-19 patients in the frontline with limited protection (Devakumar et al., 2020). These factors are in line with the results obtained in the present investigation. In particular, participants highlighted as barriers to adherence to measures and advice: living in small houses with large families, socioeconomic burden, fear of losing jobs, and working in frontline jobs in healthcare and as taxi drivers.

Further, participants described the barriers at cultural level that lead to lower adherence to measures and advice, namely collectivistic cultures, especially those from non-European countries. Cultural beliefs have a great impact on the perception, understanding, and presentation of disease and the adoption of protective behaviors on the individuals and communities (Singer et al., 2016). Researchers have investigated the role of cultural practices in the spread of disease, such as greeting styles, household size and social practices (Mayer et al., 2020). These gain importance if the beliefs derive from a collectivistic culture, in which people are cohesively integrated from birth and throughout their lives. There is a collective identity, group solidarity, emotional interdependence, sharing, duties and obligations, group decisions and the need for stable and predetermined friendships and hierarchy (Hofstede, 1980).

A study on social interaction from before the pandemic found that immigrants had more frequent contact with relatives and reported closer bonds to more people compared to the general population (Vrålstad and Wiggen, 2017). Participants in our study have similarly mentioned expectations related to their cultural practices, that is, large extended families and shared decision making, supporting each other and mutual interdependence, with the obligation to attend cultural events in order to avoid conflicts. These have acted as barriers to adherence to advice on social distancing and to avoiding large gatherings and visits. A notable finding of our study at the cultural level that acted as a barrier to adherence to measures and advice was generational differences, where older people were more oriented towards the culture of their home country and were less integrated into mainstream society and adhered less to measures and advice. The younger informants in the study showed frustration at this and struggled to balance their knowledge of and desire to adhere to measures and advice, with expectations to accept what older relatives want and think is right.

Besides facing barriers at individual and cultural level, our study participants faced challenges at the systemic level, such as poor translation of information into their respective languages, late dissemination in immigrant-specific areas, and a large amount of information subject to frequent modification. Similar findings have been reported by a study conducted in Denmark that narrated the challenges faced by immigrants in understanding the measures and advice, even though the written material was translated into 19 languages (Krusaa et al., 2020). In another study, immigrants stated that they face barriers in staying informed about COVID-19 infection control measures and advice. They are unable to understand the messages and advice coming from the authorities, such as regular press briefings, and there are shortcomings in the authorities’ capacity to make the information available in a timely manner in immigrant-specific areas. This lack of understanding and keeping up with the frequent changes in information created uncertainty, frustration and lack of trust among the participants (Brønholt et al., 2021).

While systemic barriers may affect all individuals to some extent, those specific to immigrant backgrounds—such as limited language skills, varying levels of societal knowledge, and diminished trust in institutional systems—intersect with these broader barriers, creating distinct challenges. According to intersectional theory (Atewologun, 2018), overlapping factors such as ethnicity, immigration status, and socioeconomic status intersect to compound the impact of systemic obstacles. In our study, material conditions were framed by participants as individual factors, reflecting their personal situations, such as employment status and family dynamics, rather than broader societal or systemic issues. However, we acknowledge that these individual conditions are frequently shaped by structural forces, including immigration policies, socioeconomic inequalities, and institutional access. Thus, while material barriers were categorized at the individual level based on participant narratives, their origins and impacts are deeply intertwined with structural factors, highlighting the complex interplay between individual and systemic influences.

This study has some limitations that should be noted. The study included the perspective of only a few immigrant groups and the findings should not be generalized to all immigrant groups, as we do not consider immigrants to form a homogenous group. Also, the findings derive from self-reported data, which may be influenced by social desirability bias, where participants might provide responses, they believe align with public health recommendations and therefore we cannot take the perceptions as a direct expression of an underlying truth. The findings should accordingly be used with caution, depending on the context. Further, the study does not account for the potential impact of differences in participants' socioeconomic status, immigration status, or access to healthcare, all of which could significantly affect their ability to adhere to infection control measures and may have influenced their responses during the interviews. Lastly, the dynamic and evolving nature of the COVID-19 pandemic could mean that participants' views and adherence behaviors might change over time as the public health situation and government policies continue to shift. This limits the ability to capture the full spectrum of participants' experiences during different phases of the pandemic.

Despite these limitations, the study provides valuable insights into how immigrants perceive barriers to adherence to infection control measures. These findings have several important implications for public health strategies. To improve adherence, health authorities should consider tailoring infection control communication and measures to the specific needs and contexts of diverse immigrant communities. This could involve adapting messaging to different cultural perspectives, ensuring access to information in multiple languages, and collaborating with trusted community leaders to deliver health messages. Additionally, addressing structural barriers such as work-related constraints, crowded living conditions, or limited access to healthcare would enhance the effectiveness of infection control efforts among immigrant populations.

## 5 Conclusion

The study findings give us a better understanding of how immigrants experience and perceive barriers when it comes to adhering to COVID-19 infection control measures and advice. Language and information barriers and material barriers were found at an individual level and included overcrowded households, vulnerable work situations, use of public transport and frontline jobs. Expectations relating to collectivistic cultural practice contributed to the barriers at a cultural level. Moreover, generational differences also reinforced barriers to adherence; older people were less adherent and took the pandemic less seriously, and younger people deferred to the expectations of the older adults in the family. Participants were further frustrated over the poor translation and late dissemination of information, and overload and frequent modification of information represented the barriers at a systemic level. Hence, we argue that the insight from participants is timely, and the knowledge gained can broaden the perspectives of policy makers and health authorities, and aid in improving the translation and dissemination of information among different immigrant groups and improving the socioeconomic gradient within this population. Future research might also investigate enablers and adopt a strength-based approach to identify factors that facilitate adherence among immigrant groups. This could involve examining successful strategies and positive experiences to tailor communication activities effectively, enhance engagement, and leverage community strengths to improve adherence to infection control measures. Additionally, exploring how these enablers vary across different immigrant subgroups could provide more nuanced insights for designing targeted public health interventions.

## Funding

This study was funded by Norwegian Ministry of Integration and Education.

## CRediT authorship contribution statement

**Prabhjot Kour:** Conceptualization, Data curation, Formal analysis, Writing – original draft, Writing – review & editing, Validation. **Gunnar Saebo:** Formal analysis, Methodology, Validation, Writing – review & editing. **Kristin Buvik:** Formal analysis, Methodology, Validation, Writing – review & editing. **Janne Scheffels:** Formal analysis, Methodology, Validation, Writing – review & editing. **Øystein Vedaa:** Funding acquisition, Methodology, Project administration, Resources, Validation, Writing – review & editing. **Thor Indseth:** Funding acquisition, Methodology, Project administration, Resources, Validation, Writing – review & editing.

## Declaration of competing interest

The authors declare that they have no known competing financial interests or personal relationships that could have appeared to influence the work reported in this paper.

## Figures and Tables

**Table 1:** Demographic characteristics of participants.

Variables	Number
Total participants	55
Country of Origin	
Afghanistan	7
Bosnia/Serbia	6
Iraq	6
Pakistan	6
Poland	6
Somalia	6
Sri Lanka	6
Turkey	6
Gender	
Women	31
Men	24
Age, years	
21 to 50	40
51 to 77	15
Length of residence in Norway, years	
Born in Norway	7
1 to 10	16
11 to 25	15
26 to 48	17
